# Fructose in perspective

**DOI:** 10.1186/1743-7075-10-45

**Published:** 2013-07-01

**Authors:** Richard D Feinman, Eugene J Fine

**Affiliations:** 1 State University of New York Downstate Medical Center, Brooklyn, NY, USA; 2 Albert Einstein College of Medicine, Bronx, NY, USA

**Keywords:** Carbohydrate, Fructose, Glucose, Sugar, Low-carbohydrate diet, Metabolic syndrome

## Abstract

Whether dietary fructose (as sucrose or high fructose corn syrup) has unique effects separate from its role as carbohydrate, or, in fact, whether it can be considered inherently harmful, even a toxin, has assumed prominence in nutrition. Much of the popular and scientific media have already decided against fructose and calls for regulation and taxation come from many quarters. There are conflicting data, however. Outcomes attributed to fructose — obesity, high triglycerides and other features of metabolic syndrome — are not found in every experimental test and may be more reliably caused by increased total carbohydrate. In this review, we try to put fructose in perspective by looking at the basic metabolic reactions. We conclude that fructose is best understood as part of carbohydrate metabolism. The pathways of fructose and glucose metabolism converge at the level of the triose-phosphates and, therefore, any downstream effects also occur with glucose. In addition, a substantial part of ingested fructose is turned to glucose. Regulation of fructose metabolism *per se*, is at the level of substrate control — the lower K_m_ of fructokinase compared to glucokinase will affect the population of triose-phosphates. Generally deleterious effects of administering fructose alone suggest that fructose metabolism is normally controlled in part by glucose. Because the mechanisms of fructose effects are largely those of a carbohydrate, one has to ask what the proper control should be for experiments that compare fructose to glucose. In fact, there is a large literature showing benefits in replacing total carbohydrate with other nutrients, usually fat, and such experiments sensibly constitute the proper control for comparisons of the two sugars. In terms of public health, a rush to judgement analogous to the fat-cholesterol-heart story, is likely to have unpredictable outcome and unintended consequences. Popular opinion cannot be ignored in this problem and comparing fructose to ethanol, for example, is without biochemical correlates. Also, nothing in the biochemistry suggests that sugar is a toxin. Dietary carbohydrate restriction remains the best strategy for obesity, diabetes and metabolic syndrome. The specific contribution of the removal of fructose or sucrose to this effect remains unknown.

## Introduction

Fructose (as sucrose or high fructose corn syrup (HFCS)) has become an obsession in nutrition. Painted as uniquely harmful [1], even a toxin, in both popular [2] and scientific publications [3-5], there are calls for regulation and taxation from many quarters. While there is little disagreement about the benefits of reducing sugar as a means of restricting caloric intake or of reducing total carbohydrates, especially for children, few studies directly test the effect of fructose reduction. The unique effects of adding fructose (compared to glucose) are less clear-cut (e.g. [6]) and must be reconciled with basic metabolic processes. In our opinion, the discussion of fructose shows a lack of restraint that is reminiscent of what is now recognized as a rush to judgment on dietary fat and cholesterol. It seems worthwhile to step back and put fructose in perspective.

From an evolutionary standpoint, fructose, like all carbohydrates, was a sign of good times. It is generally believed that the background availability of carbohydrates and fructose in particular, was low and intermittent in paleolithic times. Fluctuations in dietary scarcity and abundance were likely much greater than they are now. Selective advantage lay in dealing with the lean years, metabolizing whatever low background fructose was available, as well as being able to handle the high levels of consumption that frequently attend sudden good luck. It is likely that what might be considered over-consumption today was part of our behavioral repertoire early on and advantage lay in accumulating food stores, as fat and glycogen, that might not be available in the immediate future. It is important to remember that evolution is value-free and being able to eat a lot at one sitting might be beneficial in some circumstances. In general, what is called pathology is a human perspective and not a true biological concept; biological systems survive by adapting to different conditions.

In this review, we try to describe how human metabolism deals with high fructose input. Eating beyond immediate needs, for any macronutrient, predicts storage in the form of fat or glycogen and high fructose input is associated with both. Glycogen synthesis, *de novo* lipogenesis (DNL) and TAG synthesis are, in fact, characteristic of high fructose input. It is in the context of an intermittent and highly reinforcing food source, not the context of a poison, that fructose should be considered.

The metabolism of fructose is closely tied to that of glucose (Reviews: [7-10]). In this communication, we try to address the following questions: How can unique effects of fructose, increased TAG and insulin resistance, when they are observed, be reconciled with the continuity of glucose and fructose metabolic pathways? What accounts for specific effects of fructose administered parenterally? Insofar as fructose and glucose are tied to similar pathways, what is the proper control for an experiment in which one sugar is substituted for the other?

Our conclusion is that fructose is best understood as part of the general pathways of carbohydrate metabolism. Any unique effects of fructose are mediated by interactions with glucose as well as by a significant conversion of fructose to glucose. The important difference in the processing of the two sugars lies in the early steps, the much greater affinity of fructokinase compared to glucokinase, that is, regulation is at the level of substrate control, affecting the population of the triose-phosphates. Because of their close connection, replacing dietary fructose with glucose as a therapeutic measure is expected to be variable, dependent on the particular conditions and individual responses. Even those studies that show unique effects tend to have large errors and many cannot distinguish between the effects of added fructose *vs* glucose. In addition, some experimental studies, probably most, require fairly high levels of fructose. Replacing fructose with glucose is also expected to be less effective in reducing risk factors than substitution of fat for any carbohydrate. Dietary carbohydrate restriction remains the most effective, if under-utilized strategy against obesity, diabetes and metabolic syndrome in trials of various duration and protocols ([11-18] and Additional file 1).

From a public health standpoint, the scarcity of studies showing significant improvement of metabolic abnormalities by specific reduction in dietary sucrose or fructose is of concern since that is being recommended for the population at large. It might be prudent to avoid another grand experiment on the whole population — as the diet-heart paradigm has been described — in the absence of data from even small studies and without consideration of unintended consequences.

### General perspective on fructose metabolism

“Ay, in the catalogue ye go for men;

As hounds and greyhounds, mongrels, spaniels, curs,

Shoughs, water-rugs and demi-wolves, are clept

All by the name of dogs: the valued file

Distinguishes the swift, the slow, the subtle,

The housekeeper, the hunter, every one

According to the gift which bounteous nature

Hath in him closed”

— William Shakespeare, *Macbeth*

**Figure 1:**
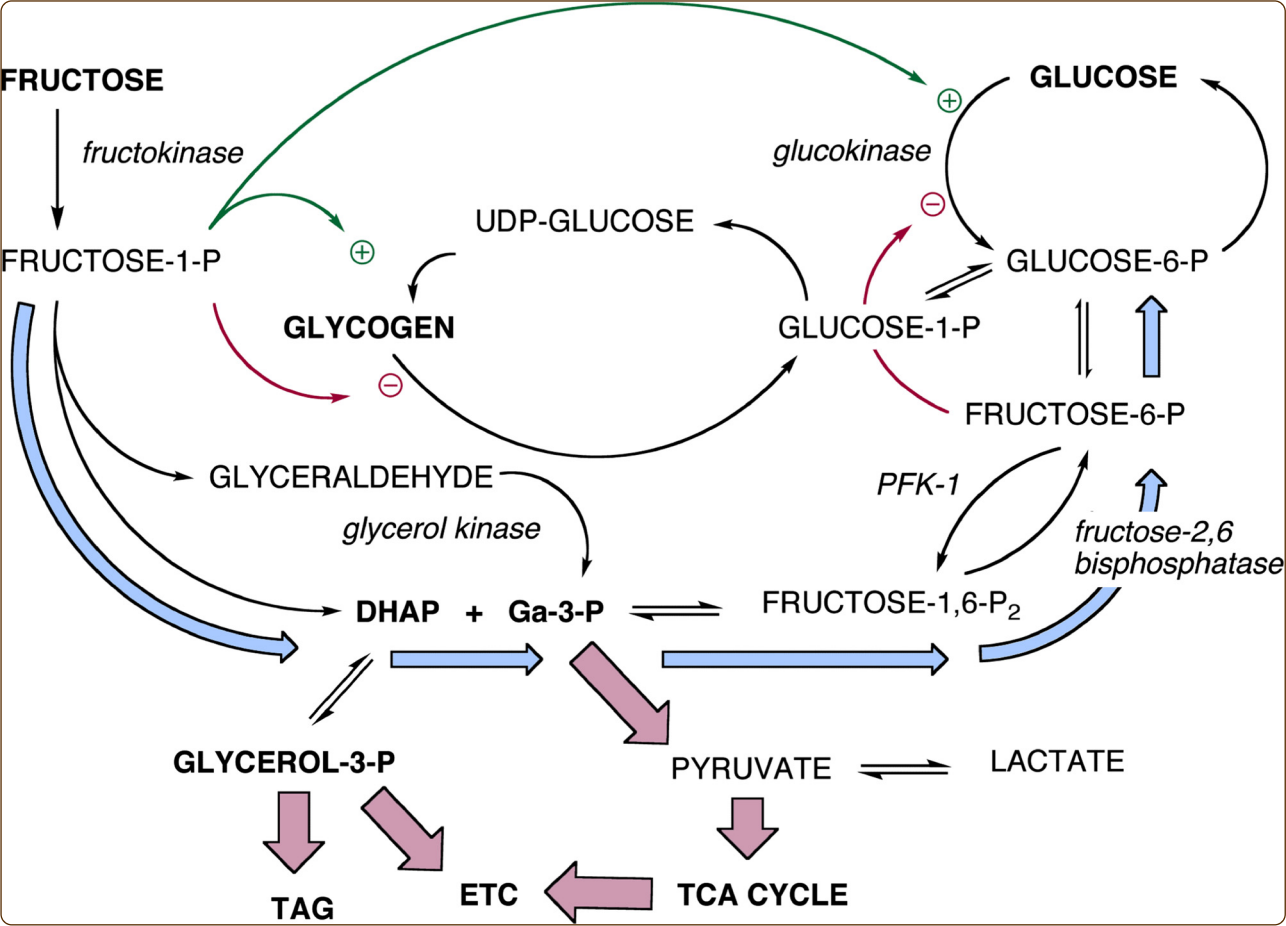
**Overview of the major aspects of hepatic metabolism of glucose and fructose.** Key points emphasized in the text: the two sugars converge at the level of the triose-phosphates (dihydroxyacetone-phosphate (DHAP) and glyceraldehyde-3-P (Ga-3-P)). Conversion of fructose to the triose-phosphates is unidirectional but fructose-1-phosphate is a positive effector of glucokinase and regulates glycogen synthesis by activating the synthase and inhibiting phosphorylase. The last effect may be different between species. Light blue arrows show the path of gluconeogenesis from fructose leading to glucose-6-P which, in turn, can produce glucose or be incorporated into glycogen.

Fructose is a carbohydrate. It is processed by incorporation into carbohydrate metabolism. Specific effects that are brought about by fructose elevation derive from an increase in the intermediates of carbohydrate metabolism. For humans, there is a background of dietary intake in which glucose almost always exceeds fructose and where fructose is rapidly cleared while glucose is maintained at constant levels. Most important, a significant amount of ingested fructose is converted to glucose (Figure 1).

Fructose and glucose metabolism converge at the level of the triose-phosphates (Figure 1). The major concerns in fructose metabolism — synthesis of glycerol-3-phosphate (glycerol-3-P) for triglyceride synthesis, generation of acetyl-CoA for the TCA cycle and *de novo* lipogenesis (DNL) — derive from these intermediates. The conundrum for understanding fructose metabolism is how a carbon in the triose-phosphates knows whether it came from fructose or glucose. The triose-phosphates are also intermediates in gluconeogenesis; under normal conditions, between 30 and 50% of ingested fructose is turned to glucose [8] and high fructose also stimulates glycogen production [19,20]. Finally, glucose is the major secretagogue of insulin which controls glycogen metabolism, triglyceride assembly and breakdown, and DNL. Taken together, these considerations suggest some circumspection in the consideration of what, if anything, “added fructose” might mean.

In such a complex system, experimental details become important. In particular, the effect of adding fructose *vs* glucose to a diet whose background composition is already high in carbohydrate is sensibly different from one where there is low total carbohydrate. Specific effects of fructose likely represent kinetic effects — more rapid population of intermediates from fructose — but these may also depend on individual conditions.

In a hypercaloric diet, as blood glucose exceeds the high K_m_ of glucokinase, the effects of added glucose or fructose will be indistinguishable. McDevitt, *et al.*[21]*,* for example, found the same level of DNL in obese and lean women exposed to 50% overfeeding of glucose or sucrose. At low sugar/low carbohydrate ingestion, the output may be controlled by compounds that derive from fructose but, in fact, it has been suggested that gluconeogenesis may be the primary effect [7]. Comparisons of low fructose and glucose are not well studied, presumably because they are not considered to involve a health risk.

### What is the appropriate control for a fructose-glucose comparison?

Metabolism is value-free, that is, the response to overconsumption of fructose may have been advantageous for an organism with intermittent supply of carbohydrate. The current discussion in nutrition, however, is couched in terms of good and bad. A person with metabolic syndrome may already have a poor response to the widely studied (and recommended) 55% carbohydrate that is considered standard [22,23] but that level is slightly higher even than current consumption. Under these conditions, any added fructose may be worse than glucose but this is expected to be a minor perturbation in the effect of the high carbohydrate that has already caused, e.g., high triglycerides.

To some extent, the question is a logical one. Numerous studies have demonstrated the benefits of restriction of carbohydrates in general (Supplement). The question, then, is what is the appropriate control for those experiments in which different carbohydrates are compared. If there is a null hypothesis, it is that the effects of fructose are primarily due to its role as a carbohydrate. The appropriate comparison, then, would be experiments in which carbohydrates across the board are substituted with another macronutrient, generally fat. We will provide an example of the effects of studies in the lab of Jeff Volek that can be compared to fructose studies [12,14,24].

### Hepatic uptake of glucose and fructose

The liver is not the only organ that metabolizes fructose but hepatic metabolism accounts for at least half of the total and represents the focus of most concern. As a kind of command center in metabolism, the liver distributes energy to other cells in the form of glucose, lactate and triglycerides although other tissues, intestines, kidney and muscle, can also process fructose directly. In the case of the kidney, lactate and glucose from fructose may also be exported.

Glucokinase, the hepatic hexokinase, has a high K_m_ — in the range of normal blood glucose — so that liver and blood glucose are in equilibrium. Hepatic glucose will fluctuate according to high blood glucose and other acute changes, for example, following starvation (Figure 1). Unlike the hexokinases of peripheral tissues, glucokinase is not subject to product inhibition by glucose-6-P but is subject to other regulation including an increase in activity in response to even small amounts of fructose.

Fructose is a poor substrate for glucokinase and most enters metabolism via the fructokinase-catalyzed reaction whose product is fructose-1-P). The low K_m_ of fructokinase (0.5 mM) means that plasma fructose is rapidly cleared. Fructokinase exists in two isoforms, A and C. The latter, expressed primarily in the liver, has high affinity for fructose and leads to rapid incorporation and depletion of ATP at least as measured in mice. Fructokinase A has wider tissue distribution and its lower affinity for fructose appears to reduce the amount of fructose for metabolism in the liver [25]. The apparent heterotropic positive cooperativity of glucokinase turns out to be due to activation by fructose-1-P (Figure 1).

### Regulation of hepatic fructose processing

Phosphorylation of fructose is regulated at the substrate level (low K_m_) and in transcription (expression is regulated by ChREBP (carbohydrate response element binding protein) [26]). There is no allosteric or hormonal control in the reaction itself and fructose is usually said to bypass phosphofructokinase-1 (PFK-1) which regulates glucose metabolism. In addition, the lysis step catalyzed by aldolase-B differs from the aldolase in glycolysis, producing dihydroxyacetone phosphate (DHAP) and glyceraldehyde. The latter, unlike the products of aldolase, must be phosphorylated. Little attention is usually paid to the kinase reaction but it points to the role of the liver as a direct consumer of fructose. Aldolase B reaction is reversible but the glyceraldehyde kinase is not: there is no “fructoneogenesis”.

Although the fructokinase reaction is not subject to allosteric regulation, the PFK-1 step itself exerts control on the down-stream metabolism of fructose. Under conditions of high energy charge and high fructose intake (the focus of current interest), PFK-1 is down regulated by ATP, and by a long feedback loop from citrate. Similarly, fructose-1, 6-bisphosphatase is *stimulated* by the high energy charge. The overall effect is increased gluconeogenesis from the triose-phosphates, (light blue pathway in Figure 1), net conversion of fructose to glucose and, as described below, a high potential for glycogen synthesis.

### Regulation of uptake and phosphorylation

**Figure 2:**
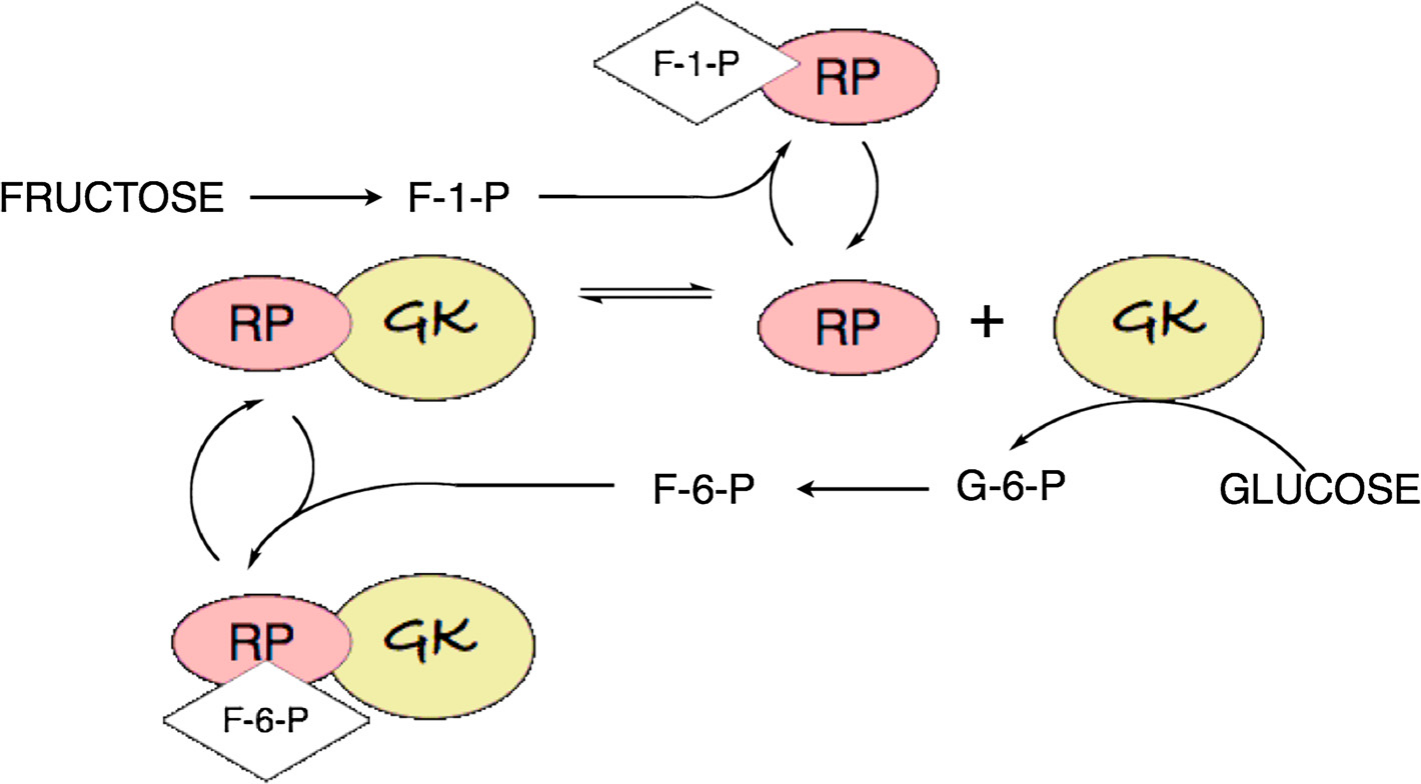
**Control of glucokinase. Binding of glucokinase-regulatory protein (RP) reduces the activity of glucokinase (GK).** Fructose-1-P relieves the inhibition by stabilizing the dissociated RP, while F-6-P furthers inhibition by stabilizing the complex. Not shown in the figure: the GK-RP complex is transported to the nucleus [27]. Dissociation leads to transport *from* the nucleus.

Fructose-1-P exerts positive allosteric control on glucokinase. It is now understood that the high K_m_ of glucokinase, and its apparent allosteric properties, are due to binding of an inhibitory glucokinase-regulatory protein (RP; Figure 2) that reduces its affinity for substrate. Fructose-1-phosphate relieves inhibition by binding to RP thereby stabilizing the dissociated proteins. Inhibition is a consequence of translocation of the glucokinase-RP complex to the nucleus [27]. Dissociation of RP and transport from the nucleus is enhanced by postprandial glucose and insulin [28]. Additional regulation is provided by fructose-6-P from glycolysis which acts as a feedback inhibitor of glucokinase, changing the affinity for RP, re-establishing binding and returning the enzyme to the nucleus, thereby creating an apparent higher K_m_ state.

Fructose might be thought of as a metabolic signal for affluence, calling for additional glucose *via* relief of inhibition of glucokinase. This response, an apparent equalizing of added fructose and glucose, may be of general importance. Administration of fructose alone may be a distinctly abnormal state for the human liver which evolved in an environment where the sugars were always presented together. Thus fructose, directly and indirectly increases the effective level of glycolytic intermediates. Hepatic carbohydrate metabolisms responds to lower plasma levels of fructose than of glucose but the combination of fructose and glucose is expected to be stronger than fructose alone. A further prediction is that under conditions of high plasma glucose, conditions where the K_m_ of glucokinase is exceeded, differences between the two sugars should cancel out. Again, DNL, under conditions of overfeeding, conform to this prediction [21].

### Gluconeogenesis

A large fraction of ingested fructose is converted to glucose [8]. Delarue, *et al*. [29] used deuterated glucose and naturally-occurring ^13^C-labelled fructose. After a 6 hour period, addition of 0.5 g/kg fructose led to the appearance of 0.27 g/kg of labeled glucose while, for ingestion of 1 g/kg fructose, 0.59 g/kg of glucose was synthesized. These values represent 56% and 59% of the fructose loads. Similar results, reviewed by Sun [8], have been found by others. Gluconeogenesis from fructose is generally assumed to proceed as shown by the light blue arrows in Figure 1[8]. It is not excluded that gluconeogenesis goes through pyruvate although the cycle, PEP → pyruvate → PEP is more energetically unfavorable than the reverse-aldolase reaction. The process will also be sensitive to the varying energy charge and presence of other macronutrients.

### Glycogen

**Figure 3:**
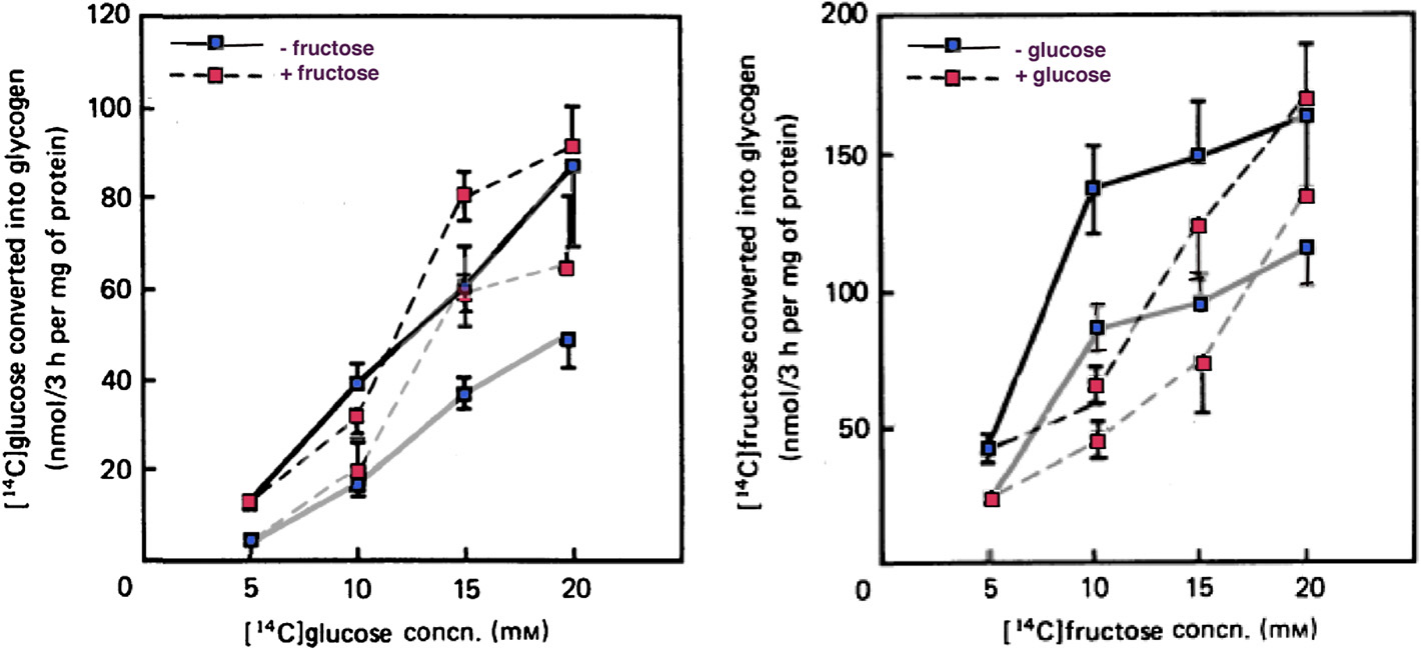
**Incorporation of labeled glucose or fructose as a function of concentration of the labeled sugar and effect of presence of other (cold) sugar.** Incorporation of labeled sugar in the absence of the other is shown by the blue symbol and the unbroken gray line. When the other (unlabeled) sugar is added there is a shift in the incorporation as shown by the red symbol and the gray broken line). All the effects are enhanced upon addition of insulin (heavy black lines). Figure redrawn from Parniak & Kalant [19].

As expected for a response to times of plenty, high fructose enhances glycogen storage (Figure 1) either directly from glucose or *via* gluconeogenesis. The interacting effects of glucose and fructose were studied in primary cultures of hepatocytes from rats deprived of food for 24 hours. Parniak and Kalant [19] measured the incorporation into glycogen of [^14^C]-glucose in the presence of cold fructose and incorporation of [^14^C]-fructose in the presence of cold glucose. The results, shown in Figure 3 indicate that label from either sugar (in the form of glucose) is incorporated into glycogen in a dose-dependent manner. Each sugar enhanced the incorporation of the other (red symbols) and, in both cases, high insulin (heavy line) increased the total yield compared to its absence (gray line). Fructose leads to greater incorporation presumably due, again, to relative higher uptake and phosphorylation but most notable is the similarity of the patterns and the extent of conversion of fructose to glucose.

This stimulating effect of fructose was demonstrated in humans by Petersen, *et al.*[20] in elegant experiments measuring the rates of hepatic glycogen synthesis *in vivo* using ^13^C-NMR under euglycemic hyperinsulinemic conditions. Infusions of ^13^C-labeled glucose in the presence or absence of fructose were followed by a cold chase to determine rates of synthesis and breakdown. In distinction to the results with hepatocytes, increased flux was due to the glycogen synthase reaction (2.5-fold) rather than an inhibition of phosphorylase activity (Figure 1).

### Insulin resistance and metabolic syndrome

**Figure 4:**
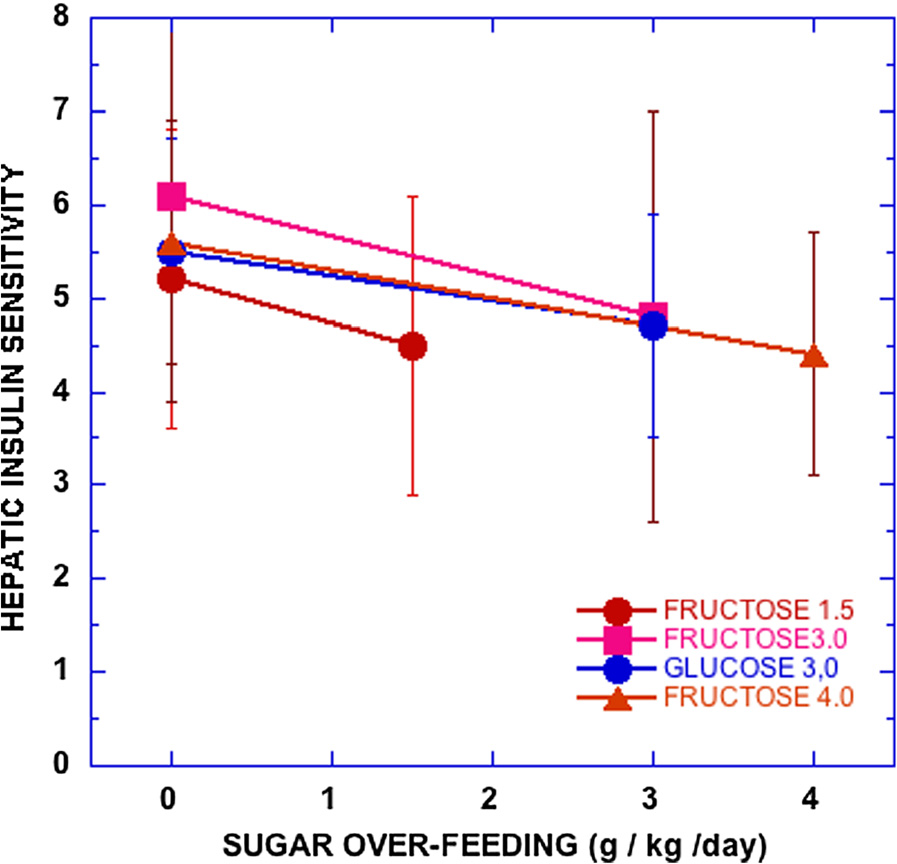
**Hepatic insulin sensitivity (100/(glucose production × fasting insulin)).** Male subjects consumed weight maintenance diets for 6–7 days and then the indicated amount of sugar was added to the same diet. Data from reference [30].

High fructose is frequently described as causing insulin resistance and metabolic syndrome (MetS) and there are several observations supporting the idea (e.g., [23]). Most such experiments, however, are done under conditions of high total carbohydrate where, again, separate effects of the sugars and their interactions are inadequately addressed. Also, not all tests show a clear fructose effect. Most recently, Lecoultre, et al. [30] demonstrated reduction in hepatic insulin sensitivity by overfeeding fructose. However, as seen in Figure 4, there was great variation and overfeeding glucose had a similar effect.

An important question is the operational definition of insulin resistance. Dirlewanger, *et al.*[31] measured the insulin required to maintain an 8 mM glucose concentration during a hyperglycemic clamp with or without the addition of 16.7 μM/kg/min fructose. The fructose addition caused an increase of insulin demand by 2.3-fold and doubled glycogen synthesis. Using radio-labeled glucose, they showed that there was no effect of fructose on endogenous glucose production or total output compared to control. When measurements were made under a hyperinsulinemic hyperglycemic clamp protocol, fructose addition caused an 11.1 μM/kg/min increase in total glucose output, increased net endogenous glucose production and increased glucose cycling. The results were described as hepatic insulin resistance, that is, more insulin was needed to maintain the level of blood glucose in the presence of fructose and, conversely, high insulin did not repress production of glucose.

Although the results conformed to an operational definition of insulin resistance, Dirlewanger, *et a*l. [31] provided an explanation of the results which seems like a normal response to the experimental conditions and which would be consistent with the mechanism in Figure 1: the *effective* hepatic glucose concentration is very much higher in the fructose group due to the extensive gluconeogenesis. This will lead to enhanced total glucose output despite the high insulin concentration, an insulin concentration that was able to repress output in the glucose-alone controls. At the same time, fructose-1-P, by causing an increased glucokinase activity, would lead to enhanced re-uptake of glucose and increased glucose cycling. Thus, apparent insulin resistance is the effect of higher levels of glucose intermediates in a fructose-stimulated cell and does not represent any basic detrimental change in hepatic physiology.

The concept of insulin sensitivity or resistance is thus strongly dependent on the method of measurement and the operational definition. Insulin resistance may be a characteristic of type 2 diabetes and the *sine qua non* of metabolic syndrome but, depending on what is measured, need not be associated with maladaptive responses to food or other stimulation and my represent productive adaptation to different conditions.

Again looking at the influence of total dietary carbohydrate as a control, it is known that carbohydrate restriction across the board will improve all of the features of MetS. It was suggested, in fact, that the response of all of the individual markers of MetS to reduced carbohydrate might serve as an operational definition [13], given that there is some question as to whether the syndrome even exists [32]. This is important in that it is reasonable that at least some part of insulin resistance is down-regulation of response due to chronic high insulin as in many hormonal systems. Reduction in glucose, rather than fructose, will reduce this high insulin.

A critical prospective test was carried out by Volek and coworkers who assigned 40 overweight men and women with the atherogenic dyslipidemia characteristic of MetS (high TAG, low HDL-C, high concentration of small dense LDL) to a very low carbohydrate ketogenic diet (VLCKD) in [12,14,24]. An isocaloric fat-restricted diet served as control. Compared to these controls, the VLCKD group showed greater improvement in body mass, in glycemic control, and in many of the features of MetS: TAG, HDL-C, apo B, apo A-1 and LDL particle size distribution as well as a greater anti-inflammatory effect. The extent to which these effects of carbohydrate restriction were specifically due to removing fructose or sucrose is unknown but the magnitude of the effect (although in the direction of improvement) are generally larger than those seen in glucose-fructose comparisons.

The point here is that we know that restricting carbohydrates is effective in reducing the features of metabolic syndrome. Until there is evidence that it is specifically sugar that was removed, logic dictates that carbohydrate restriction is the preferred approach.

### Triglycerides

Carbohydrate-induced hypertriglyceridemia and its flip-side, reduction in triglycerides by dietary carbohydrate restriction, are well established phenomena. The effect is generally attributed to the increased flux of insulin which inhibits lipolysis and the reduction in glycerol-3-P to provide substrate for synthesis. It is unknown whether and to what extent there is a unique effect of fructose but several papers have indicated a high potential for fructose to increase plasma TAG compared to the effect of glucose [22,33-35]. There are, however, conflicting reports [11] and, again, most of the recent studies have been done against a baseline of high total carbohydrate or unusually high fructose, or both, meaning that baseline may already be very high.

Chong, *et al.* showed that, under acute postprandial conditions, little activity from radio-labeled fructose showed up in the fatty acid portion of TAG, that is, fatty acids are primarily from endogenous fat [35]. DNL is relatively small for either glucose or fructose. On the other hand, a maor part (38%) of the glycerol comes from labeled fructose, consistent with the idea that the main effect of fructose is to more rapidly, or to a greater extent, populate the triose-phosphates.

**Figure 5:**
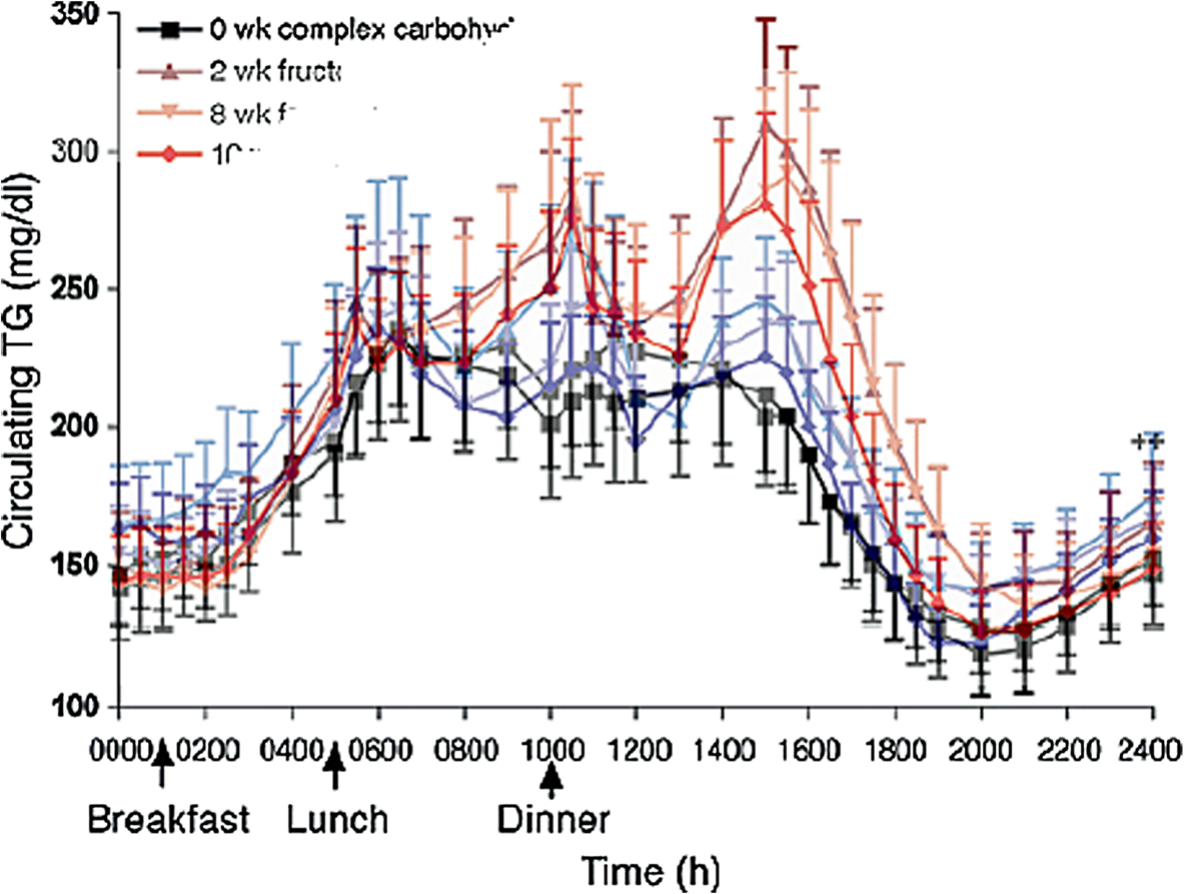
**24-hour circulating triacylglycerol before (black) or after 2, 8 and 10 weeks of consuming glucose-sweetened (blue) or fructose-sweetened beverages (red) providing 25% of energy.** Superposition of Figures 2A and 2B, redrawn from Stanhope, et al. [34].

Figure 5 shows typical results from Stanhope, *et al.*[34] who measured the relative effects of consumption of glucose- or fructose-sweetened beverages in overweight and obese subjects. The sugar-sweetened drinks provided 25% of energy requirements for 10 weeks. The figure shows superimposed fructose and glucose curves from reference [34]. On average, the fructose curve has larger postprandial excursions but the difference in the curves is of the order of 10% which, in combination with the very large error bars makes the data questionable. The error bars in the figure, in fact, represent the standard error of the mean (SEM). SEM can provide a statistical measure of the difference in the populations, but in terms of presentation of data, it does not communicate very well a sense of the true variability of the individual data. The standard deviation (SD), a better indicator of the true variation, is obtained by multiplying by √n which, in this case, is 3.7 for glucose and 4.1 for fructose. With such large variation, a couple of outliers would change the character of the curve. In other words, whereas there is a unique effect of fructose, it is not large and appears to be highly variable.

**Figure 6:**
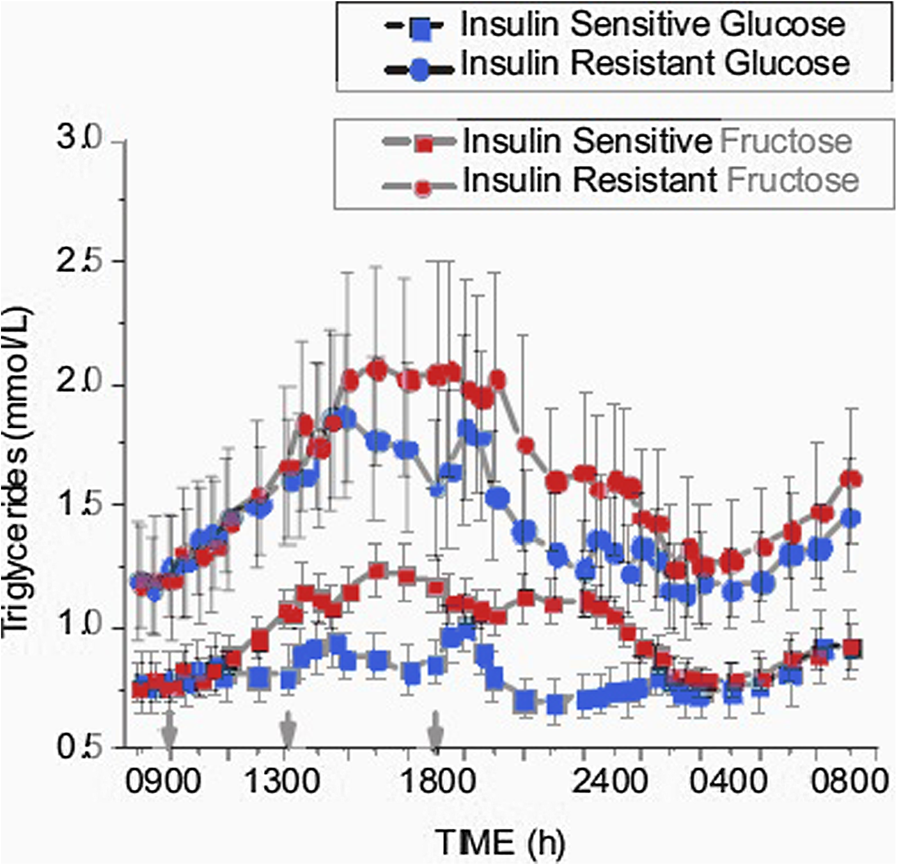
**Comparative effects of fructose-sweetened (red) and glucose-sweetened beverages (blue).** Data from Teff, et al. [5] were merged. Insulin-sensitive (−□-) and insulin-resistant (−○-) sub-groups were separately analyzed from the original populations.

Similarly, Teff, et al. [22] found significantly higher TAG in a group that consumed 30% of their total energy input in fructose-sweetened beverages as compared to a group who ingested glucose-sweetened beverages. This level of consumption is fairly high compared to the average in the United States (about 10%) but, of greater significance is, once again, the large individual variation. Dividing subjects in both groups into sub-populations on the basis of insulin sensitivity showed that, in fact, differences due to insulin sensitivity within each sugar were as great as the differences between the two sugars. As shown 0in Figure 6, insulin-resistant subjects in the glucose arm had higher TAG than insulin-sensitive subjects in the fructose arm. Because of the importance of insulin resistance, it is reasonable to think that insulin will be the true variable of interest.

The association between levels of dietary carbohydrate and plasma TAG may be the single most predictable effect of nutrients on lipid metabolism (reviews: [11,13,14]). That increases in plasma TAG are not always seen in high fructose feeding [36] suggests that it is not the major, or at least, not the only player. The proper control, again, is substitution of total carbohydrate with something else, sensibly fat. Although no such explicit comparison has been done, replacing total carbohydrate with fat always shows much greater changes than experiments in which sugars are exchanged. For example, Volek, *et al*. [24] compared two hypocaloric diets (~1,500 kcal): a carbohydrate-restricted diet (%carbohydrate: fat:protein = 12:59:28) and a low-fat diet (LFD) (56:24:20) in 40 subjects with atherogenic dyslipidemia (high triglycerides, low HDL, high concentration of small dense LDL).

**Figure 7:**
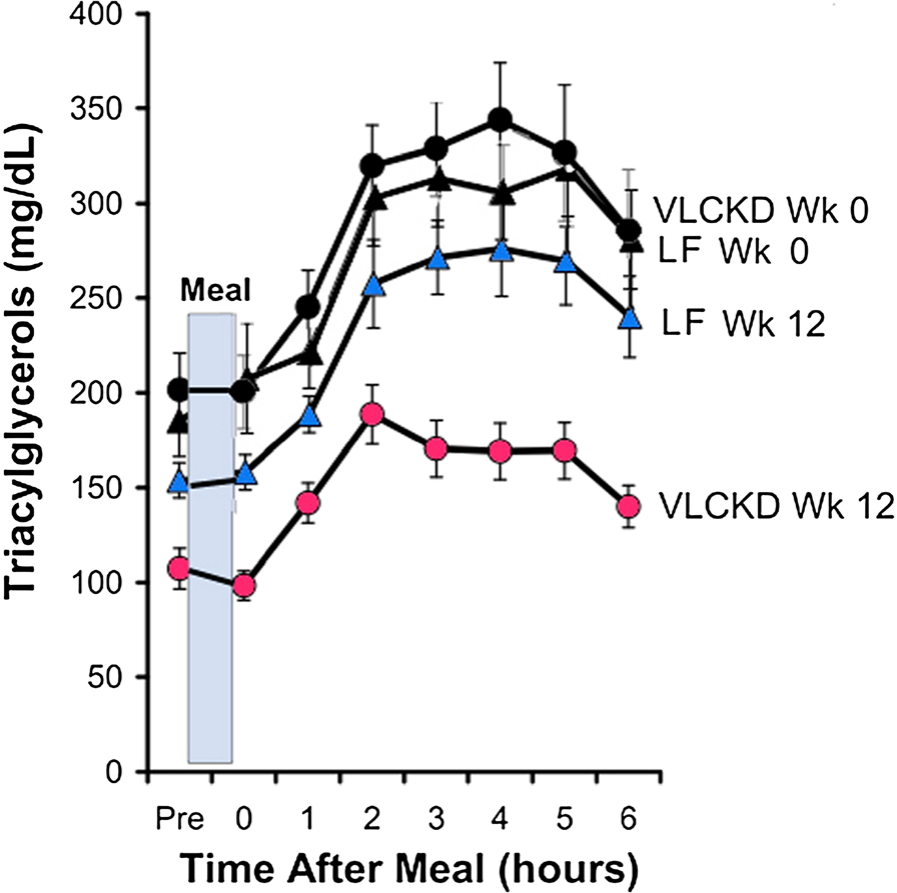
**Effect of diet on postprandial lipemic responses in subjects with atherogenic dyslipidemia.** Absolute TAG values in subjects who consumed a very low carbohydrate ketogenic diet (VLCKD) or a low-fat diet (LF) for 12 weeks. Redrawn from Volek, et al. [24].

Figure 7 from Volek, *et al.* is representative. Changes in TAG from replacing total carbohydrate are larger and more reliable than studies in which glucose replaces fructose. Hollenbeck [36] reviewed studies through 1993 on the effect of fructose on lipid metabolism. Of 18 relevant studies, she considered that only 8 met the criteria of 1) sufficient dietary and experimental control, 2) glucose or starch for comparison; and 3) had limited heterogeneity present in the study population. Of these 8, half showed no change in plasma TAG while one found no change in a normal group but an increase in subjects with hypertriglyceridemia, and one study similarly found no change in the normal population with increases in subjects with hyperinsulinemia. Only two studies found increases in TAG.

### *De novo* lipogenesis

**Figure 8:**
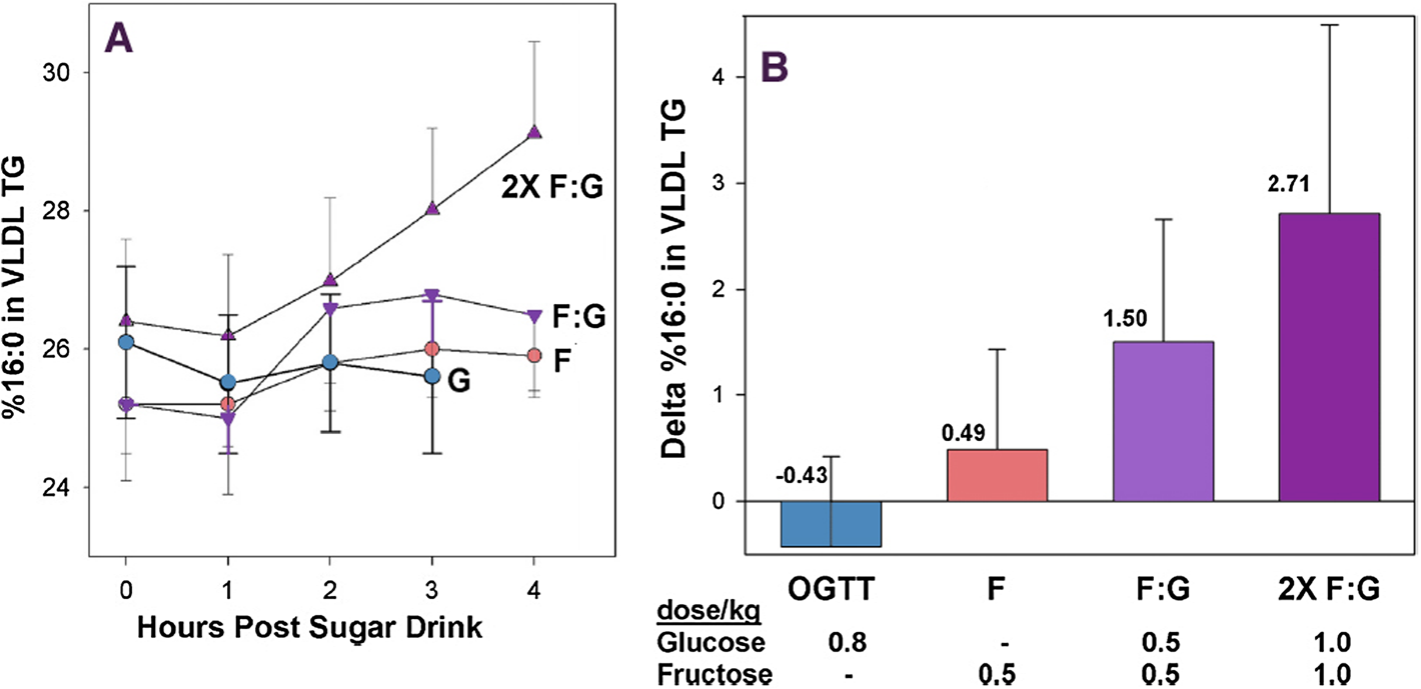
**Effect of sugar on percentage of palmitate (16:0) in VLDL TG before and after OGTT (glucose, 75 g; average, 0.8 g/kg) and indicated levels of fructose (F) and glucose (G).** Redrawn from reference [38].

*De novo* lipogenesis (DNL), is an important feature of the state of high energy charge and/or high carbohydrate. Differential effects of glucose and fructose (reviewed in reference [37]) follow the pattern described here. For example, Hudgins, *et al.*[38] measured percent increase in palmitate in VLDL-TAG. Consistent with the idea that fructose ingestion may change total availability of sugars in the liver, they found that fructose has a much greater effect than glucose when administered alone; an oral glucose tolerance test (OGTT) had little effect. However, adding glucose to a dose of fructose increased DNL, and as total carbohydrate becomes high, exceeding the K_m_ of glucokinase, there is little difference between sugars (Figure 8), absolute changes, however, are small and, again, there is great variability.

One of the features of Volek’s study described above was that, despite a 3-fold higher intake of dietary saturated fat during the VLCKD, saturated fatty acids in TAG and cholesteryl ester were significantly decreased compared to low-fat controls. That this was due to a decrease in DNL was shown by a corresponding reduction in palmitoleic acid (16:1n-7), the product of the desaturase. Palmitoleic acid is present in only low concentrations in the diet and is generally taken as an indication of DNL.

### Effects of pure fructose

Because of its limited effect on insulin secretion, it was originally thought that fructose might be a desirable sugar for people with diabetes. However, this proved to be not only ineffective but led to the risk of lactic acidosis. Similar effects of pure fructose are observed in parenteral nutrition [39] or administering fructose during exercise [40]. This response has traditionally been explained as a kinetic effect due to the rapid phosphorylation of fructose and a depletion of ATP. One of the consequences is increased glycolysis and increased lactic acid production. Under conditions where both fructose and glucose are available, there is somewhat greater lactic acid production from higher fructose although there is no threat of acidosis and lactic acid is one of the ways that fructose supplies energy to extrahepatic cells. While speculative, a reasonable deduction is that hepatic metabolism has evolved so as to require glucose for fructose metabolism. The cytosol is much more oxidizing than the mitochondrion and conversion of DHAP to glycerol-3-P requires NADH. At low NADH, glyceraldehyde-3-P will be oxidized to provide ATP and NADH, resulting in conversion to pyruvate and to lactate production. In addition, there are reported digestive effects of fructose alone which indicate the involvement of problems in absorption. It may be that results with fructose alone cannot be compared to results where both sugars are present and such interventions may not be a good model for human consumption which almost never includes pure fructose.

### Effect of energy charge

It is widely reported that fructose depletes ATP due to the fructokinase reaction [1] although this has generally been observed in isolated hepatocyte cultures and with addition of pure fructose. The major focus of current interest are studies done under conditions of high energy charge and the reactions characteristic of fructose — glycogen and triglyceride formation — require ATP. Veech, et *al.* showed that high ATP accompanied a high sucrose diet [41]. Oddly, Abdelmalek, *et al.*[42] reported that fructose lowered hepatic ATP as measured by ^31^P-nuclear magnetic resonance (NMR) but their data did not support that deduction. The effects on ATP may be dependent on particular conditions. The effects of different conditions of substrate source and particularly exercise are beyond the scope of this review but there is, again, a good deal of variability as seen even in the effects on rates of oxidation of different sugars (Review: [8]). As noted above, the presence of fructose tends to increase the levels of plasma lactate but, generally, differences between dietary glucose *vs*. fructose + glucose tends to become smaller and, as in other conditions, fructose alone is not desirable [43].

## Conclusions

There are clearly specific effects of fructose but we emphasize that these must be rationalized in the face of the continuum between fructose and glucose metabolism. Control of fructose metabolism is primarily at the level of substrate regulation, the more favorable K_m_ of fructokinase compared to glucokinase. Because downstream metabolism of fructose from the triose-phosphates is the same as that for glucose, there is an expectation that there will be variability among studies. This expectation is borne out and even those that have clear-cut outcomes, show significant statistical error.

Finally, nobody is suggesting that continued high consumption of sugar is good but there there is a logical problem and a practical problem. Logically, you cannot say that we will look at the effect of fructose but not the effect of carbohydrates. It doesn’t make sense.

Removing sugar without replacement is obviously good for weight loss but practically speaking, if we want to reduce sugar consumption isocalorically, we must consider whether to replace sugar with starch or with another nutrient, usually fat. There are numerous studies showing the benefit of the latter approach but few demonstrating the value of the former. Showing that fructose is worse than glucose under some conditions is not the same thing as showing that specifically removing fructose is beneficial. Until these comparisons are made, it seems like a good idea to keep some perspective.

While studies with combinations of fructose and glucose are consistent with a general effect of carbohydrate, fructose alone appears to have aberrant behavior and one might speculate that the system evolved to deal with the two sugars together, consistent with the general absence of pure fructose outside of experimental trials.

From the perspective of ideas in the popular media, however, there is little relation between fructose metabolism and ethanol metabolism and it is unreasonable to refer to fructose as a toxin.

## Discussion

The strongest argument for caution in a strategy of specifically removing fructose (as sucrose or HFCS) from the food supply is the absence of significant prospective trials. In terms of basic metabolism, fructose is incorporated into general carbohydrate metabolism which may have specifically evolved to deal with the sudden appearance of desirable food. Persistence in a state of over-consumption has serious consequences and there is a clear benefits in restricting sugar, especially for children. However, suggesting that fructose is somehow a foreign substance is not consistent with the science and, therefore, should not be the basis of policy. There is a continuum from scientific studies to popular media that suggests a circumspect approach is unlikely and, in our opinion, there is a clear sense of a rush to judgement on sugar, entirely analogous to that in the diet-heart-cholesterol phenomenon. Perhaps the most important similarity is that both official agencies and individual doctors and researchers are recommending, even demanding, reduction in sugar, despite the absence of any experimental test of the idea. The message to reduce fat and cholesterol was, similarly, made before any test of what the outcomes might be. Given increasing evidence of risk from high total carbohydrate intake, the likelihood of unintended consequences from reducing fructose alone (starch replacing sugar) is strong.

Emphasizing fructose outside of general carbohydrate metabolism has the serious limitation of substantially ignoring the hormonal effects of glucose the major secretagogue of insulin. In people with type 2 diabetes, removing starch is more beneficial than removing sugar [44] and effective treatment has been demonstrated in several studies from Nuttall and Gannon where the controlling variable is reduction of what the authors call bioavailable glucose [17,18,45]. In this area, at least, it would be good to proceed carefully.

A virtue of the current emphasis on the dangers of fructose is the appeal to an analysis based on basic metabolism [3,4]. The points made in the current review should be included in that analysis.

## Abbreviations

ChREBP: Carbohydrate response element binding protein; DNL: *de novo* lipogenesis, Ga-3-P, Glyceraldehyde-3-P; glycerol-3-P: Glycerol-3-phosphate; HFCS: High fructose corn syrup; LF: Low-fat; OGTT: Oral glucose tolerance test; PFK-1: Phosphofructokinase-1; TAG: Triacylgylcerol; VLCKD: Very low carbohydrate ketogenic diet.

## Competing interests

The authors have received grant support from the Veronica and Robert C. Atkins Foundation. RDF has written popular articles for several publications including Diabetes Health, Muscular Development and for Fleishman-Hillard, Inc. whose client is the American Corn Refiners Association.

## Supplementary Material

Additional file 1 Feinman_&_Fine_Additional_Material. Click here for file
