# Supplementary material for: Fructose in perspective
Source: Nutr Metab (Lond). 2013 Jul 1;10:45. doi: 10.1186/1743-7075-10-45 (PMC3708747; doi:10.1186/1743-7075-10-45)
Supplement: Additional file 1 — Feinman_&_Fine_Additional_Material. [file 1743-7075-10-45-S1.pdf]

Bibliography of low-carbohydrate studies.

1. Accurso A, Bernstein RK, Dahlqvist A, Draznin B, Feinman RD, Fine EJ, Gleed A, Jacobs DB, Larson G, Lustig RH *et al*: **Dietary carbohydrate restriction in type 2 diabetes mellitus and metabolic syndrome: time for a critical appraisal**. *Nutr Metab (Lond)* 2008, **5**(1):9.
2. Al-Sarraj T, Saadi H, Volek JS, Fernandez ML: **Carbohydrate restriction favorably alters lipoprotein metabolism in Emirati subjects classified with the metabolic syndrome**. *Nutr Metab Cardiovasc Dis* 2010, **20**(10):720-726.
3. Al-Sarraj T, Saadi H, Volek JS, Fernandez ML: **Metabolic syndrome prevalence, dietary intake, and cardiovascular risk profile among overweight and obese adults 18-50 years old from the United Arab Emirates**. *Metab Syndr Relat Disord* 2010, **8**(1): 39-46.
4. Austin GL, Dalton CB, Hu Y, Morris CB, Hankins J, Weinland SR, Westman EC, Yancy WS, Jr., Drossman DA: **A Very Low-Carbohydrate Diet Improves Symptoms and Quality of Life in Diarrhea-Predominant Irritable Bowel Syndrome**. *Clin Gastroenterol Hepatol* 2009.
5. Austin GL, Dalton CB, Hu Y, Morris CB, Hankins J, Weinland SR, Westman EC, Yancy WS, Jr., Drossman DA: **A very low-carbohydrate diet improves symptoms and quality of life in diarrhea-predominant irritable bowel syndrome**. *Clin Gastroenterol Hepatol* 2009, **7**(6):706-708 e701.
6. Austin GL, Thiny MT, Westman EC, Yancy WS, Jr., Shaheen NJ: **A very low-carbohydrate diet improves gastroesophageal reflux and its symptoms**. *Dig Dis Sci* 2006, **51**(8):1307-1312.
7. Austin MA, Hokanson JE, Edwards KL: **Hypertriglyceridemia as a cardiovascular risk factor**. *Am J Cardiol* 1998, **81**(4A):7B-12B.
8. Baum JI, Layman DK, Freund GG, Rahn KA, Nakamura MT, Yudell BE: **A reduced carbohydrate, increased protein diet stabilizes glycemic control and minimizes adipose tissue glucose disposal in rats**. *J Nutr* 2006, **136**(7):1855-1861.
9. Boden G, Sargrad K, Homko C, Mozzoli M, Stein TP: **Effect of a low-carbohydrate diet on appetite, blood glucose levels, and insulin resistance in obese patients with type 2 diabetes**. *Ann Intern Med* 2005, **142**(6):403-411.
10. Dashti HM, Al-Zaid NS, Mathew TC, Al-Mousawi M, Talib H, Asfar SK, Behbahani AI: **Long term effects of ketogenic diet in obese subjects with high cholesterol level**. *Mol Cell Biochem* 2006, **286**(1-2):1-9.
11. Dashti HM, Bo-Abbas YY, Asfar SK, Mathew TC, Hussein T, Behbahani A, Khoursheed MA, Al-Sayer HM, Al-Zaid NS: **Ketogenic diet modifies the risk factors of heart disease in obese patients**. *Nutrition* 2003, **19**(10):901-902.
12. Dashti HM, Mathew TC, Hussein T, Asfar SK, Behbahani A, Khoursheed MA, Al-Sayer HM, Bo-Abbas YY, Al-Zaid NS: **Long-term effects of a ketogenic diet in obese patients**. *Exp Clin Cardiol* 2004, **9**(3):200-205.

13. Dashti HM, Mathew TC, Khadada M, Al-Mousawi M, Talib H, Asfar SK, Behbahani AI, Al-Zaid NS: **Beneficial effects of ketogenic diet in obese diabetic subjects**. *Mol Cell Biochem* 2007.
14. Cornier MA, Donahoo WT, Pereira R, Gurevich I, Westergren R, Enerback S, Eckel PJ, Goalstone ML, Hill JO, Eckel RH *et al*: **Insulin sensitivity determines the effectiveness of dietary macronutrient composition on weight loss in obese women**. *Obes Res* 2005, **13**(4):703-709.
15. Feinman RD, Fine EJ, Volek JS: **Analysis of dietary interventions. A simple payoff matrix for display of comparative dietary trials**. *Nutr J* 2008, **7**(1):24.
16. Feinman RD, Volek JS: **Carbohydrate restriction as the default treatment for type 2 diabetes and metabolic syndrome**. *Scand Cardiovasc J* 2008, **42**(4):256-263.
17. Feinman RD, Volek JS: **Low carbohydrate diets improve atherogenic dyslipidemia even in the absence of weight loss**. *Nutr Metab (Lond)* 2006, **3**:24.
18. Feinman RD, Volek JS, Westman E: **Dietary Carbohydrate Restriction in the Treatment of Diabetes and Metabolic Syndrome**. *Clinical Nutrition Insight* 2008, **34(12)**:1-5.
19. Feinman RD, Westman EC, Volek JS: **Low carbohydrate and low fat diets in diabetes, cardiovascular disease and metabolic syndrome**. *Cellscience Reviews* 2006, **3**(1).
20. Forsythe CE, Phinney SD, Feinman RD, Volk BM, Freidenreich D, Quann E, Ballard K, Puglisi MJ, Maresh CM, Kraemer WJ *et al*: **Limited effect of dietary saturated fat on plasma saturated fat in the context of a low carbohydrate diet**. *Lipids* 2010, **45**(10): 947-962.
21. Forsythe CE, Phinney SD, Fernandez ML, Quann EE, Wood RJ, Bibus DM, Kraemer WJ, Feinman RD, Volek JS: **Comparison of low fat and low carbohydrate diets on circulating Fatty Acid composition and markers of inflammation**. *Lipids* 2008, **43**(1): 65-77.
22. Champ CE, Volek JS, Siglin J, Jin L, Simone NL: **Weight gain, metabolic syndrome, and breast cancer recurrence: are dietary recommendations supported by the data?** *International journal of breast cancer* 2012, **2012**:506868.
23. Layman DK, Boileau RA, Erickson DJ, Painter JE, Shiue H, Sather C, Christou DD: **A reduced ratio of dietary carbohydrate to protein improves body composition and blood lipid profiles during weight loss in adult women**. *J Nutr* 2003, **133**(2):411-417.
24. Layman DK, Shiue H, Sather C, Erickson DJ, Baum J: **Increased dietary protein modifies glucose and insulin homeostasis in adult women during weight loss**. *J Nutr* 2003, **133**(2):405-410.
25. Foster GD, Wyatt HR, Hill JO, Makris AP, Rosenbaum DL, Brill C, Stein RI, Mohammed BS, Miller B, Rader DJ *et al*: **Weight and metabolic outcomes after 2 years on a low-carbohydrate versus low-fat diet: a randomized trial**. *Ann Intern Med* 2010, **153**(3):147-157.
26. Foster GD, Wyatt HR, Hill JO, McGuckin BG, Brill C, Mohammed BS, Szapary PO, Rader DJ, Edman JS, Klein S: **A randomized trial of a low-carbohydrate diet for obesity**. *N Engl J Med* 2003, **348**(21):2082-2090.

27. Noakes M, Foster PR, Keogh JB, James AP, Mamo JC, Clifton PM: **Comparison of isocaloric very low carbohydrate/high saturated fat and high carbohydrate/low saturated fat diets on body composition and cardiovascular risk**. *Nutr Metab (Lond)* 2006, **3**:7.
28. Noakes M, Foster P, Keogh J, Clifton P: **Very low carbohydrate diets for weight loss and cardiovascular risk**. *Asia Pac J Clin Nutr* 2004, **13**(Suppl):S64.
29. Samaha FF, Foster GD, Makris AP: **Low-carbohydrate diets, obesity, and metabolic risk factors for cardiovascular disease**. *Curr Atheroscler Rep* 2007, **9**(6):441-447.
30. Gannon MC, Hoover H, Nuttall FQ: **Further decrease in glycated hemoglobin following ingestion of a LoBAG30 diet for 10 weeks compared to 5 weeks in people with untreated type 2 diabetes**. *Nutr Metab (Lond)* 2010, **7**:64.
31. Gannon MC, Nuttall FQ: **Control of blood glucose in type 2 diabetes without weight loss by modification of diet composition**. *Nutr Metab (Lond)* 2006, **3**:16.
32. Gannon MC, Nuttall FQ: **Effect of a high-protein, low-carbohydrate diet on blood glucose control in people with type 2 diabetes**. *Diabetes* 2004, **53**(9):2375-2382.
33. Gannon MC, Nuttall FQ, Grant CT, Ercan-Fang S, Ercan-Fang N: **Stimulation of insulin secretion by fructose ingested with protein in people with untreated type 2 diabetes**. *Diabetes Care* 1998, **21**(1):16-22.
34. Gannon MC, Nuttall FQ, Saeed A, Jordan K, Hoover H: **An increase in dietary protein improves the blood glucose response in persons with type 2 diabetes**. *Am J Clin Nutr* 2003, **78**(4):734-741.
35. Gannon MC, Nuttall FQ, Westphal SA, Fang S, Ercan-Fang N: **Acute metabolic response to high-carbohydrate, high-starch meals compared with moderate-carbohydrate, low-starch meals in subjects with type 2 diabetes**. *Diabetes Care* 1998, **21**(10):1619-1626.
36. Feinman RD: **Fad diets in the treatment of diabetes**. *Current diabetes reports* 2011, **11**(2):128-135.
37. Feinman RD, Fine EJ: **"A Calorie is a calorie" violates the second law of thermodynamics.** *Nutrition Journal* 2004, **3**(9).
38. Feinman RD, Fine EJ: **Nonequilibrium thermodynamics and energy efficiency in weight loss diets**. *Theor Biol Med Model* 2007, **4**:27.
39. Feinman RD, Fine EJ: **Thermodynamics and Metabolic Advantage of Weight Loss Diets.** *Metabolic Syndrome and Related Disorders* 2003, **1**:209-219.
40. Feinman RD, Fine EJ: **Whatever happened to the second law of thermodynamics?** *Am J Clin Nutr* 2004, **80**(5):1445-1446; author reply 1446.
41. Feinman RD, Makowske M: **Metabolic Syndrome and Low-Carbohydrate Ketogenic Diets in the Medical School Biochemistry Curriculum.** *Metabolic Syndrome and Related Disorders* 2003, **1**:189-198.
42. Feinman RD, Vernon MC, Westman EC: **Low carbohydrate diets in family practice: what can we learn from an internet-based support group**. *Nutr J* 2006, **5**:26.
43. Fine EJ, Feinman RD: **Thermodynamics of weight loss diets**. *Nutr Metab (Lond)* 2004, **1**(1):15.

44. Karam J, Nessim F, McFarlane S, Feinman R: **Carbohydrate Restriction and Cardiovascular Risk**. *Current Cardiovascular Risk Reports* 2008, **2**(2):88-94.
45. Mavropoulos JC, Buschemeyer WC, 3rd, Tewari AK, Rokhfeld D, Pollak M, Zhao Y, Febbo PG, Cohen P, Hwang D, Devi G *et al*: **The effects of varying dietary carbohydrate and fat content on survival in a murine LNCaP prostate cancer xenograft model**. *Cancer Prev Res (Phila)* 2009, **2**(6):557-565.
46. Mavropoulos JC, Yancy WS, Hepburn J, Westman EC: **The effects of a low-carbohydrate, ketogenic diet on the polycystic ovary syndrome: a pilot study**. *Nutr Metab (Lond)* 2005, **2**:35.
47. Tendler D, Lin S, Yancy WS, Jr., Mavropoulos J, Sylvestre P, Rockey DC, Westman EC: **The effect of a low-carbohydrate, ketogenic diet on nonalcoholic fatty liver disease: a pilot study**. *Dig Dis Sci* 2007, **52**(2):589-593.
48. Golay A, Allaz AF, Morel Y, de Tonnac N, Tankova S, Reaven G: **Similar weight loss with low- or high-carbohydrate diets**. *Am J Clin Nutr* 1996, **63**(2):174-178.
49. Golay A, Eigenheer C, Morel Y, Kujawski P, Lehmann T, de Tonnac N: **Weight-loss with low or high carbohydrate diet?** *Int J Obes Relat Metab Disord* 1996, **20**(12):1067-1072.
50. Nuttall FQ, Gannon MC: **The metabolic response to a high-protein, low-carbohydrate diet in men with type 2 diabetes mellitus**. *Metabolism* 2006, **55**(2):243-251.
51. Nuttall FQ, Gannon MC, Saeed A, Jordan K, Hoover H: **The metabolic response of subjects with type 2 diabetes to a high-protein, weight-maintenance diet**. *J Clin Endocrinol Metab* 2003, **88**(8):3577-3583.
52. Nuttall FQ, Schweim K, Hoover H, Gannon MC: **Effect of the LoBAG30 diet on blood glucose control in people with type 2 diabetes**. *Br J Nutr* 2008, **99**(3):511-519.
53. Nuttall FQ, Schweim K, Hoover H, Gannon MC: **The Metabolic Effect of a LoBAG30 Diet in Men With Type 2 Diabetes**. *Am J Physiol Endocrinol Metab* 2006.
54. Phinney SD: **Ketogenic diets and physical performance**. *Nutr Metab (Lond)* 2004, **1**(1):2.
55. Phinney SD, Bistrian BR, Evans WJ, Gervino E, Blackburn GL: **The human metabolic response to chronic ketosis without caloric restriction: preservation of submaximal exercise capability with reduced carbohydrate oxidation**. *Metabolism* 1983, **32**(8):769-776.
56. Phinney SD, Davis PG, Johnson SB, Holman RT: **Obesity and weight loss alter serum polyunsaturated lipids in humans**. *Am J Clin Nutr* 1991, **53**(4):831-838.
57. Nielsen JV, Joensson E: **Low-carbohydrate diet in type 2 diabetes. Stable improvement of bodyweight and glycemic control during 22 months follow-up**. *Nutr Metab (Lond)* 2006, **3**:22.
58. Nielsen JV, Jonsson E, Ivarsson A: **A low carbohydrate diet in type 1 diabetes: clinical experience--a brief report**. *Ups J Med Sci* 2005, **110**(3):267-273.
59. Nielsen JV, Jonsson E, Nilsson AK: **Lasting improvement of hyperglycaemia and bodyweight: low-carbohydrate diet in type 2 diabetes. A brief report**. *Ups J Med Sci* 2005, **110**(2):179-183.
60. Nielsen JV, Westerlund P, Bygren P: **A low-carbohydrate diet may prevent end-stage renal failure in type 2 diabetes. A case report**. *Nutr Metab (Lond)* 2006, **3**:23.

61. Tay J, Brinkworth GD, Noakes M, Keogh J, Clifton PM: **Metabolic effects of weight loss on a very-low-carbohydrate diet compared with an isocaloric high-carbohydrate diet in abdominally obese subjects**. *J Am Coll Cardiol* 2008, **51**(1): 59-67.
62. Nordmann AJ, Nordmann A, Briel M, Keller U, Yancy WS, Jr., Brehm BJ, Bucher HC: **Effects of low-carbohydrate vs low-fat diets on weight loss and cardiovascular risk factors: a meta-analysis of randomized controlled trials**. *Arch Intern Med* 2006, **166**(3):285-293.
63. Lofgren I, Zern T, Herron K, West K, Sharman MJ, Volek JS, Shachter NS, Koo SI, Fernandez ML: **Weight loss associated with reduced intake of carbohydrate reduces the atherogenicity of LDL in premenopausal women**. *Metabolism* 2005, **54**(9): 1133-1141.
64. Mutungi G, Torres-Gonzalez M, McGrane MM, Volek JS, Fernandez ML: **Carbohydrate restriction and dietary cholesterol modulate the expression of HMG-CoA reductase and the LDL receptor in mononuclear cells from adult men**. *Lipids Health Dis* 2007, **6**:34.
65. Ruano G, Windemuth A, Kocherla M, Holford T, Fernandez ML, Forsythe CE, Wood RJ, Kraemer WJ, Volek JS: **Physiogenomic analysis of weight loss induced by dietary carbohydrate restriction**. *Nutr Metab (Lond)* 2006, **3**:20.
66. Sharman MJ, Fernandez ML, Zern TL, Torres-Gonzalez M, Kraemer WJ, Volek JS: **Replacing dietary carbohydrate with protein and fat decreases the concentrations of small LDL and the inflammatory response induced by atherogenic diets in the guinea pig**. *J Nutr Biochem* 2008.
67. Sharman MJ, Gomez AL, Kraemer WJ, Volek JS: **Very low-carbohydrate and low-fat diets affect fasting lipids and postprandial lipemia differently in overweight men**. *J Nutr* 2004, **134**(4):880-885.
68. Sharman MJ, Kraemer WJ, Love DM, Avery NG, Gomez AL, Scheett TP, Volek JS: **A ketogenic diet favorably affects serum biomarkers for cardiovascular disease in normal-weight men**. *J Nutr* 2002, **132**(7):1879-1885.
69. Sharman MJ, Volek JS: **Weight loss leads to reductions in inflammatory biomarkers after a very-low-carbohydrate diet and a low-fat diet in overweight men**. *Clin Sci (Lond)* 2004, **107**(4):365-369.
70. Torres-Gonzalez M, Leite JO, Volek JS, Contois JH, Fernandez ML: **Carbohydrate restriction and dietary cholesterol distinctly affect plasma lipids and lipoprotein subfractions in adult guinea pigs**. *J Nutr Biochem* 2008.
71. Volek JS, Sharman MJ, Gomez AL, Scheett TP, Kraemer WJ: **An Isoenergetic Very Low-Carbohydrate Diet Is Associated With Improved Serum High-Density Lipoprotein Cholesterol (HDL-C), Total Cholesterol to HDL-C Ratio, Triacylglycerols, and Postprandial Lipemic Responses Compared to a Low-Fat Diet in Normal Weight, Normolipidemic Women**. *J Nutr* 2003, **133**(9):2756-2761.
72. Volek JS, Feinman RD: **Carbohydrate restriction improves the features of Metabolic Syndrome. Metabolic Syndrome may be defined by the response to carbohydrate restriction**. *Nutr Metab (Lond)* 2005, **2**:31.

73. Volek JS, Fernandez ML, Feinman RD, Phinney SD: **Dietary carbohydrate restriction induces a unique metabolic state positively affecting atherogenic dyslipidemia, fatty acid partitioning, and metabolic syndrome**. *Prog Lipid Res* 2008, **47**(5):307-318.
74. Volek JS, Forsythe CE: **The case for not restricting saturated fat on a low carbohydrate diet**. *Nutr Metab (Lond)* 2005, **2**:21.
75. Volek JS, Forsythe CE: **Very-Low-Carbohydrate Diets**. In: *Essentials of Sports Nutrition and Supplements.* edn. Edited by Antonio J, Kalman D, Stout JR, Greenwood M, Willoughby DS, Haff GG. Totowa, NJ: Humana Press; 2008.
76. Volek JS, Phinney SD, Forsythe CE, Quann EE, Wood RJ, Puglisi MJ, Kraemer WJ, Bibus DM, Fernandez ML, Feinman RD: **Carbohydrate Restriction has a More Favorable Impact on the Metabolic Syndrome than a Low Fat Diet**. *Lipids* 2009, **44**(4):297-309.
77. Volek JS, Sharman MJ, Gomez AL, DiPasquale C, Roti M, Pumerantz A, Kraemer WJ: **Comparison of a very low-carbohydrate and low-fat diet on fasting lipids, LDL subclasses, insulin resistance, and postprandial lipemic responses in overweight women**. *J Am Coll Nutr* 2004, **23**(2):177-184.
78. Volek JS, Sharman MJ, Gomez AL, Judelson DA, Rubin MR, Watson G, Sokmen B, Silvestre R, French DN, Kraemer WJ: **Comparison of energy-restricted very low-carbohydrate and low-fat diets on weight loss and body composition in overweight men and women**. *Nutr Metab (Lond)* 2004, **1**(1):13.
79. Volek JS, Sharman MJ, Gomez AL, Scheett TP, Kraemer WJ: **An isoenergetic very low carbohydrate diet improves serum HDL cholesterol and triacylglycerol concentrations, the total cholesterol to HDL cholesterol ratio and postprandial lipemic responses compared with a low fat diet in normal weight, normolipidemic women**. *J Nutr* 2003, **133**(9):2756-2761.
80. Volek JS, Westman EC: **Very-low-carbohydrate weight-loss diets revisited**. *Cleve Clin J Med* 2002, **69**(11):849, 853, 856-848 passim.
81. Volek J, Bibus D, Fernandez M, Forsythe C, Quann E, Silvestre R, Wood R, Kraemer J, Phinney S: **A hypocaloric very low carbohydrate ketogenic diet results in a greater reduction in the percent and absolute amount of plasma triglyceride saturated fatty acids compared to a low fat diet.** *NAASO, Boston, MA, October, 2006* 2006.
82. Westman EC, Feinman RD, Mavropoulos JC, Vernon MC, Volek JS, Wortman JA, Yancy WS, Phinney SD: **Low-carbohydrate nutrition and metabolism**. *Am J Clin Nutr* 2007, **86**(2):276-284.
83. Westman EC, Mavropoulos J, Yancy WS, Volek JS: **A Review of Low-carbohydrate Ketogenic Diets**. *Curr Atheroscler Rep* 2003, **5**(6):476-483.
84. Westman EC, Volek JS: **Postprandial triglycerides in response to high fat: role of dietary carbohydrate**. *Eur J Clin Invest* 2004, **34**(1):74; author reply 75.
85. Westman EC, Volek JS, Feinman RD: **Carbohydrate restriction is effective in improving atherogenic dyslipidemia even in the absence of weight loss**. *Am J Clin Nutr* 2006, **84**(6):1549; author reply 1550.
86. Westman EC, Yancy WS, Haub MD, Volek JS: **Insulin resistance from a low carbohydrate, high fat diet perspective**. *Metab Syndr Relat Disord* 2005, **3**(1):14-18.

87. Wood RJ, Volek JS, Davis SR, Dell'ova C, Fernandez ML: **Effects of a carbohydrate-restricted diet on emerging plasma markers for cardiovascular disease**. *Nutr Metab (Lond)* 2006, **3**(1):19.
88. Wood RJ, Volek JS, Liu Y, Shachter NS, Contois JH, Fernandez ML: **Carbohydrate restriction alters lipoprotein metabolism by modifying VLDL, LDL, and HDL subfraction distribution and size in overweight men**. *J Nutr* 2006, **136**(2):384-389.
89. deOgburn R, Leite JO, Ratliff J, Volek JS, McGrane MM, Fernandez ML: **Effects of increased dietary cholesterol with carbohydrate restriction on hepatic lipid metabolism in Guinea pigs**. *Comparative medicine* 2012, **62**(2):109-115.
90. Shai I, Schwarzfuchs D, Henkin Y, Shahar DR, Witkow S, Greenberg I, Golan R, Fraser D, Bolotin A, Vardi H *et al*: **Weight loss with a low-carbohydrate, Mediterranean, or low-fat diet**. *N Engl J Med* 2008, **359**(3):229-241.
91. Hickey JT, Hickey L, Yancy WS, Hepburn J, Westman EC: **Clinical Use of a Carbohydrate-Restricted Diet to Treat the Dyslipidemia of the Metabolic Syndrome**. *Metabolic Syndrome and Related Disorders* 2003, **1**(3):227-232.
92. Vernon M, Mavropoulos J, Transue M, Yancy W, Jr, Westman E: **Clinical Experience of a Carbohydrate-Restricted Diet: Effect on Diabetes Mellitus**. *Metabolic Syndrome and Related Disorders* 2003, **1**:233-237.
93. Westman EC: **A Review of Very Low Carbohydrate Diets for Weight Loss**. *JCOM* 1999, **6**(7):36-40.
94. Westman EC: **Is dietary carbohydrate essential for human nutrition?** *Am J Clin Nutr* 2002, **75**(5):951-953; author reply 953-954.
95. Westman EC, Yancy WS, Jr., Humphreys M: **Dietary treatment of diabetes mellitus in the pre-insulin era (1914-1922)**. *Perspect Biol Med* 2006, **49**(1):77-83.
96. Westman EC, Yancy WS, Jr., Olsen MK, Dudley T, Guyton JR: **Effect of a low-carbohydrate, ketogenic diet program compared to a low-fat diet on fasting lipoprotein subclasses**. *Int J Cardiol* 2006, **110**(2):212-216.
97. Westman EC, Yancy WS, Jr., Vernon MC: **Is a low-carb, low-fat diet optimal?** *Arch Intern Med* 2005, **165**(9):1071-1072.
98. Westman EC, Yancy WS, Edman JS, Tomlin KF, Perkins CE: **Effect of 6-month adherence to a very low carbohydrate diet program**. *Am J Med* 2002, **113**(1):30-36.
99. Westman EC, Yancy WS, Mavropoulos JC, Marquart M, McDuffie JR: **The Effect of a Low-Carbohydrate, Ketogenic Diet Versus a Low-Glycemic Index Diet on Glycemic Control in Type 2 Diabetes Mellitus**. *Nutr Metab (Lond)* 2008, **5**(36).
100. Yancy WS, Jr., Foy M, Chalecki AM, Vernon MC, Westman EC: **A low-carbohydrate, ketogenic diet to treat type 2 diabetes**. *Nutr Metab (Lond)* 2005, **2**:34.
101. Yancy WS, Jr., Olsen MK, Guyton JR, Bakst RP, Westman EC: **A low-carbohydrate, ketogenic diet versus a low-fat diet to treat obesity and hyperlipidemia: a randomized, controlled trial**. *Ann Intern Med* 2004, **140**(10):769-777.
102. Yancy WS, Jr., Vernon MC, Westman EC: **A Pilot trial of a Low-Carbohydrate, Ketogenic Diet in Patients with Type 2 Diabetes**. *Metabolic Syndrome and Related Disorders* 2003, **1**(3):239-243.

103. Yancy WS, Jr., Westman EC, McDuffie JR, Grambow SC, Jeffreys AS, Bolton J, Chalecki A, Oddone EZ: **A randomized trial of a low-carbohydrate diet vs orlistat plus a low-fat diet for weight loss**. *Arch Intern Med* 2010, **170**(2):136-145.
104. Yancy WS, Jr., Almirall D, Maciejewski ML, Kolotkin RL, McDuffie JR, Westman EC: **Effects of two weight-loss diets on health-related quality of life**. *Qual Life Res* 2009, **18**(3):281-289.
105. Lecheminant JD, Smith BK, Westman EC, Vernon MC, Donnelly JE: **Comparison of a reduced carbohydrate and reduced fat diet for LDL, HDL, and VLDL subclasses during 9-months of weight maintenance subsequent to weight loss**. *Lipids Health Dis* 2010, **9**:54.
106. Lecheminant JD, Gibson CA, Sullivan DK, Hall S, Washburn R, Vernon MC, Curry C, Stewart E, Westman EC, Donnelly JE: **Comparison of a low carbohydrate and low fat diet for weight maintenance in overweight or obese adults enrolled in a clinical weight management program**. *Nutr J* 2007, **6**:36.
